# Polyvinyl Alcohol-Coated Polycaprolactone Microneedles as Polymeric Microdevices for Transdermal Antigen Delivery and Immune Activation

**DOI:** 10.3390/polym18192327

**Published:** 2026-09-24

**Authors:** Aline F. Teixeira, Leandro T. Kochi, Ana Carola De La Via Reis, Jose E. Rojas, Daniele Ribeiro de Araujo, Wendel A. Alves, Ana Lucia T. O. Nascimento

**Affiliations:** 1 Laboratório de Desenvolvimento de Vacinas, Instituto Butantan, São Paulo 05503-900, SP, Brazil; 2 Centro de Ciências Naturais e Humanas, Universidade Federal do ABC, Santo André 09210-580, SP, Brazil; 3 Department of Biophysics, Universidade Federal de São Paulo, São Paulo 04021-001, SP, Brazil

**Keywords:** polymeric microneedles, polycaprolactone, poly(vinyl alcohol) coating, ex vivo skin permeation, transdermal antigen delivery, DNP-KLH

## Abstract

Conventional needle-and-syringe vaccination is associated with pain, needle-related anxiety, the need for trained personnel, and logistical challenges related to vaccine distribution and storage. Microneedle (MN) technology has emerged as a minimally invasive strategy for transdermal delivery and as a platform for functional polymeric microdevices. In this study, polycaprolactone (PCL) microneedles coated with hydrophilic polymers were evaluated as a model platform for antigen delivery. Ex vivo assays using porcine ear skin were explored as a preliminary approach to assess antigen transport from polymer-coated PCL microneedles. However, antigen-specific quantification in the receptor compartment was limited by the lack of specificity of absorbance-based measurements in this biological matrix. Scanning electron microscopy (SEM) showed that PVA coating partially altered microneedle tip morphology, whereas optical coherence tomography (OCT) and optical microscopy confirmed that PVA20%-PCL-MNs maintained the ability to insert into porcine skin and generate visible insertion sites. Mechanical compression testing further showed that polymer-coated PCL-MNs maintained resistance to axial deformation under the tested conditions. PVA20%-PCL-MNs were also non-toxic and maintained high HEK293T cell viability after 24 h. Based on their preserved insertion capacity, mechanical robustness, ability to support DNP-KLH permeation, and preliminary cytocompatibility, PVA20%-PCL-MNs were evaluated in BALB/c mice using a two-dose prime-boost regimen. Although intraperitoneal immunization induced higher IgG levels, transdermal immunization with PVA20%-PCL-MNs promoted booster-dependent antigen-specific IgG responses. IgG2a/IgG1 ratio analysis indicated a predominantly IgG1-biased humoral profile, and splenocytes from mice immunized with PVA20%-PCL-MNs produced detectable levels of IFN-γ and IL-10 after in vitro stimulation. Together, these findings support PVA-coated PCL microneedles as a proof-of-concept polymeric microdevice platform for minimally invasive antigen delivery and immunogenicity assessment.

## 1. Introduction

Vaccination represents one of the most impactful and life-saving advances in modern medicine. According to the World Health Organization, immunization prevents approximately 3.5 to 5 million deaths each year from diseases such as diphtheria, tetanus, pertussis, influenza, and measles (WHO). Nevertheless, vaccine-preventable diseases continue to cause substantial morbidity and mortality, particularly in populations with limited vaccine coverage [1]. Several factors contribute to this challenge, including difficulties associated with vaccine distribution, the need for trained personnel, and limitations of conventional needle-and-syringe administration. Parenteral injection may cause pain, discomfort, and needle-related anxiety, which can negatively affect vaccine acceptance [2]. In addition, many liquid vaccine formulations require refrigerated storage, making cold-chain maintenance costly and logistically challenging, especially in low-resource settings [3]. These limitations have encouraged the development of alternative delivery systems that may simplify vaccine administration, reduce dependence on trained personnel, and improve accessibility.

Microneedle (MN) technology has emerged as a promising alternative for minimally invasive transdermal delivery of therapeutic and immunological agents [4]. MNs consist of micron-sized projections on a supporting substrate that can penetrate the stratum corneum and deliver cargo into the epidermal and dermal layers [5,6]. These skin compartments contain antigen-presenting cells, including Langerhans cells and dermal dendritic cells, making the transdermal route particularly attractive for immunization strategies. Because the route of administration can strongly influence the quality and magnitude of immune responses, MN-based platforms have gained increasing attention as functional microdevices for antigen delivery [7].

Several types of MNs have been developed, including solid, coated, hollow, dissolving, hydrogel-forming, and polymeric MNs, each offering specific advantages for different applications [5,8]. Solid MNs are commonly used to create microchannels in the skin for subsequent drug delivery. Coated MNs carry the active compound on their microneedle surfaces, enabling rapid release upon insertion [9]. Hollow MNs contain internal channels through which liquid formulations can be delivered. Dissolving MNs are prepared from water-soluble polymers or sugars that dissolve upon insertion, releasing the encapsulated cargo. Hydrogel-forming MNs absorb interstitial fluid and swell within the skin, enabling controlled transport of therapeutic agents [10]. Polymeric MNs, in turn, provide versatile platforms for engineering mechanical properties, degradation behavior, and release profiles by selecting polymers, controlling processing conditions, and surface functionalizing [4,11].

Polycaprolactone (PCL) is a semicrystalline, hydrophobic, biodegradable, and biocompatible synthetic polymer with broad use in biomedical applications [12]. Owing to its durability, processability, and compatibility with other polymers, PCL has been explored in drug delivery systems, biodegradable implants, and tissue engineering scaffolds [13,14,15]. In addition, its low cost and ability to be processed into different geometries make PCL an attractive candidate for the fabrication of polymeric microdevices and sustainable biomedical materials [16,17]. Recently, PCL-based MNs have also been investigated as electrochemical biosensors for detecting IFN-*α*, highlighting their potential as multifunctional platforms for infectious disease diagnosis [18].

For antigen-delivery applications, combining a mechanically stable polymeric MN substrate with a hydrophilic functional coating may offer a simple strategy for enabling cargo loading and rapid release after skin insertion. In this context, polyvinyl alcohol (PVA) is particularly attractive as a coating material due to its hydrophilicity, water solubility, film-forming ability, and broad use in biomedical and pharmaceutical applications [19]. Such a design allows PCL to act as the structural microneedle platform, while the PVA coating serves as a functional polymeric interface for antigen deposition and release.

In this study, we evaluated PVA-coated PCL microneedles as a proof-of-concept polymeric platform for transdermal antigen delivery. PVA- and alginate-coated PCL-MNs were initially evaluated using ex vivo assays across porcine ear skin as a preliminary approach to investigate the feasibility of macromolecular antigen transport using dinitrophenyl-conjugated keyhole limpet hemocyanin (DNP-KLH) as a model antigen. The coated microneedles were further characterized by SEM, mechanical compression testing, OCT, and optical microscopy to assess coating morphology, mechanical robustness, skin penetration, and insertion efficiency in porcine skin. Based on their preserved functional skin insertion, mechanical performance, preliminary ex vivo assessment in porcine skin, and preliminary in vitro cytocompatibility, PVA20%-PCL-MNs were evaluated in BALB/c mice using a two-dose prime-boost immunization regimen. The platform was assessed for antigen-specific IgG production, IgG2a/IgG1 ratio, and cytokine production by splenocytes stimulated in vitro. Overall, this work investigates the feasibility of PVA20%-PCL-MNs as functional polymeric microdevices for minimally invasive antigen administration and immunogenicity assessment.

## 2. Materials and Methods

### 2.1. Manufacture of PCL Microneedles

Polydimethylsiloxane (PDMS) molds containing pyramidal cavities arranged in a 10 × 10 array were used to fabricate the microneedle arrays. Each pyramidal cavity measured 700 µm in height and 200 µm at the base, with a 500 µm pitch (Sigma-Aldrich, Darmstadt, Hesse, Germany; cat. 934577-10EA). Polycaprolactone (PCL; MW 70,000–90,000 g mol^−1^; Sigma-Aldrich, St. Louis, MO, USA; cat. 440744) was used as the base polymer for microneedle fabrication.

Briefly, three PCL pellets were deposited onto each PDMS mold and heated at 60 °C for 30 min in a glass Petri dish to melt the polymer. The molds were then centrifuged at 3612× *g* and 40 °C for 10 min to ensure complete filling of the microneedle cavities. After centrifugation, the molds were kept at room temperature for 24 h to allow polymer solidification. The resulting PCL-MNs were carefully demolded using tweezers.

### 2.2. Preparation of Polymer-Coated PCL-MNs

For antigen loading, a 20% *w*/*v* poly(vinyl alcohol) solution (PVA; Mowiol^®^ 8-88, Sigma-Aldrich, Darmstadt, Hesse, Germany; cat. 81383) was prepared in water under magnetic stirring at 80 °C for 1 h. The PVA solution was then cooled to 37 °C before antigen addition. Separately, a 4% *w*/*v* sodium alginate solution (ALG; Sigma-Aldrich, cat. W201502) was prepared in water under magnetic stirring at room temperature for 1 h.

Dinitrophenyl-conjugated keyhole limpet hemocyanin (DNP-KLH; Sigma-Aldrich, cat. 324121) was added to the corresponding polymer solution to obtain final antigen amounts of either 10 µg or 100 µg per microneedle array. The DNP-KLH solution and the polymer solution were mixed at a 1:1 *v*/*v* ratio before coating. A volume of 20 µL of the resulting polymer-antigen mixture was carefully deposited onto each PCL microneedle array to produce DNP-KLH-loaded PVA20%-PCL-MNs or DNP-KLH-loaded ALG4%-PCL-MNs. The coated microneedles were allowed to dry at room temperature before use.

The antigen dose reported for each formulation corresponds to the nominal amount of DNP-KLH deposited onto each microneedle array before drying.

### 2.3. Ex Vivo Permeation Assays Across Porcine Ear Skin

Porcine ears were obtained from a local slaughterhouse and used as an ex vivo skin model. Subcutaneous tissue was carefully removed, and the skin was separated from the underlying cartilage. After processing, porcine ear skin samples were stored at −20 °C until use. The protocol was approved by the UNIFESP Institutional Animal Care and Use Committee (CEUA #1686050526).

For permeation assays, full-thickness porcine ear skin samples with an average thickness of 1.3 ± 0.2 mm were placed in Franz diffusion cells. The donor compartment had an effective diffusion area of 0.6 cm^2^, and the receptor compartment was filled with 4.3 mL of 0.1 M sodium phosphate buffer, pH 7.4. The system was maintained under magnetic stirring at 350 rpm at 32.5 °C.

Microneedle arrays measuring 1 cm^2^ and containing 0.1 µg cm^−2^ of DNP-KLH were positioned onto the skin surface. PVA20%-PCL-MNs and ALG4%-PCL-MNs were evaluated. At predetermined time points from 0.5 to 30 h, aliquots of 0.3 mL were withdrawn from the receptor compartment and immediately replaced with the same volume of fresh buffer.

DNP-KLH concentration in the receptor medium was determined by absorbance at 280 nm using a NanoDrop 2000 spectrophotometer (Thermo Fisher, Waltham, MA, USA). Quantification was performed using a previously established calibration curve, with a slope of 0.12421 and R^2^ = 0.9867. Permeation profiles were expressed as the mean ± SD from five independent microneedle samples per composition. Flux values, J, were calculated from the linear portion of the permeation profiles and expressed as µg cm^−2^ h^−1^. Because absorbance at 280 nm is not antigen-specific and may be influenced by skin-derived components released into the receptor medium, these data were interpreted as an absorbance-based estimate of DNP-KLH-equivalent permeation rather than as definitive antigen-specific quantification.

### 2.4. Skin Penetration and Insertion Efficiency Assays

Optical coherence tomography (OCT) and optical microscopy were used to evaluate the insertion performance of uncoated PCL-MNs, PVA20%-PCL-MNs, and ALG4%-PCL-MNs in porcine skin. OCT images were acquired using a Callisto 930 nm OCT imaging system (Thorlabs, Newtown, NJ, USA). Two-dimensional cross-sectional images were obtained to assess skin surface disruption and localized indentations associated with microneedle insertion.

For insertion efficiency analysis, microneedle arrays were applied to porcine skin using a commercial applicator. An approximate force of 30 N was applied for 30 s. After removal of the microneedle arrays, the skin surface was examined by optical microscopy to identify visible insertion sites. Insertion efficiency was calculated as the percentage of visible puncture marks relative to the total number of microneedles in each array. For each formulation, 100 microneedles were evaluated.

As a complementary standardized insertion model, Parafilm^®^ M (Bemis Company Inc., Soignies, Belgium) was used to evaluate the insertion properties of the microneedle arrays under multilayer conditions. Parafilm^®^ M sheets were folded into an eight-layer film, and the microneedle arrays were inserted using a commercial applicator. After 30 s of insertion, the microneedle arrays were removed, and the number of holes formed in each Parafilm^®^ layer was determined. Insertion efficiency was expressed as the percentage of holes per layer relative to the total number of microneedles in the array. Experiments were performed in triplicate, and 100 microneedles were evaluated for each replicate.

### 2.5. Mechanical Compression Testing of Coated PCL-MNs

The mechanical behavior of uncoated PCL-MNs, PVA20%-PCL-MNs, and ALG4%-PCL-MNs was evaluated by axial compression testing using an Anton Paar MCR 502 rheometer equipped with a 25 mm parallel plate. Each microneedle array was positioned base-down on the testing stage and compressed vertically against the upper plate under controlled displacement. The top plate was lowered at a constant rate of 0.035 mm s^−1^, and force–displacement curves were recorded for each formulation. The maximum compression force was defined as the highest force recorded within the tested displacement range. When applicable, measurements were performed in triplicate, and representative force–displacement curves were used to compare the mechanical response of uncoated and polymer-coated microneedles. This analysis was used to assess whether polymer coating and coating-induced changes in tip morphology affected the mechanical robustness of the microneedle arrays.

### 2.6. Scanning Electron Microscopy Characterization

The morphology of uncoated PCL-MNs, PVA20%-PCL-MNs, and ALG4%-PCL-MNs was evaluated by scanning electron microscopy (SEM) using a Quanta 250 microscope (FEI Company, Eindhoven, The Netherlands). Before analysis, the samples were mounted on metallic stubs using conductive carbon tape and coated with a 25 nm-thick gold layer by sputter coating to reduce charging effects and improve image resolution.

SEM analyses were performed under high-vacuum conditions at accelerating voltages ranging from 10 to 20 kV. Surface morphology and microneedle tip geometry were evaluated using a secondary electron detector. Backscattered electron imaging was used when necessary to assess contrast differences associated with the coated microneedle surfaces.

### 2.7. In Vitro Cytotoxicity Evaluation of PVA20%-PCL-MNs

HEK293T (ATCC CRL-3216) cells were selected as a representative, standard cell model for initial, broad-spectrum in vitro cytotoxicity screening of the PVA20%-PCL-MNs. HEK293T cells were cultured in complete DMEM and seeded into 24-well plates at a density of 1 × 10^4^ cells per well. After overnight incubation at 37 °C in a 5% CO_2_ atmosphere, PVA20%-PCL-MNs were sterilized by UV irradiation and subsequently added to the wells for 24 h.

After incubation, the culture medium was replaced with MTT solution (0.5 mg mL^−1^) in DMEM, and the cells were incubated for 4 h under the same conditions. The resulting formazan crystals were solubilized with DMSO, and absorbance was measured at 570 nm. Cell viability was calculated relative to untreated control cells, which were considered 100% viable. Ethanol-treated cells were used as the cytotoxic control.

### 2.8. Stability Assay of DNP-KLH After Release from MNs

PVA20%-PCL-MNs containing 10 µg or 100 µg of Dinitrophenyl-conjugated keyhole limpet hemocyanin (DNP-KLH; Sigma-Aldrich, cat. 324121) were prepared as previously described. To evaluate antigen stability after release, MNs were incubated in 200 µL of 1X Dulbecco’s Phosphate-Buffered Saline (DPBS 1X, Gibco (Waltham, MA, USA), Cat. 14190-136) for 1 h at room temperature. After incubation, the released antigen solution was collected and mixed with SDS sample buffer containing β-mercaptoethanol. Samples were denatured at 96 °C for 10 min. Subsequently, samples and a protein molecular weight marker (HMW SDS Calibration Kit, GE Healthcare (Chicago, IL, USA), Cat. 17-0615-01) were loaded onto a 4–20% gradient SDS-PAGE polyacrylamide gel (Bio-Rad, Hercules, CA, USA). A concentration of DNP-KLH (4 µg) was also loaded onto a gel as a control. Electrophoresis was performed at room temperature for approximately 2 h at a constant voltage of 80 V in 1X SDS-PAGE running buffer. The gel was stained with Coomassie Brilliant Blue G-250 for 2 h, destained overnight in Coomassie destaining solution, and imaged using a document scanner.

### 2.9. Preparation of DNP-KLH-Loaded PVA20%-PCL-MNs and Immunization of Mice

For antigen loading, a 20% *w*/*v* PVA aqueous solution containing either 10 µg or 100 µg of dinitrophenyl-conjugated keyhole limpet hemocyanin (DNP-KLH; Sigma-Aldrich, St. Louis, MO, USA; cat. 324121) was prepared. A volume of 20 µL of this solution was carefully deposited onto each PCL-MN array to obtain DNP-KLH-loaded PVA20%-PCL-MNs. The coated microneedles were allowed to dry before use.

For the immunization experiments, female BALB/c mice weighing 18–22 g were randomly assigned to groups of five animals. Animals were anesthetized by intraperitoneal injection of ketamine and xylazine at doses of 100 mg kg^−1^ and 10 mg kg^−1^, respectively (Syntec, Tamboré, SP, Brazil). The dorsal region was shaved and cleaned with 70% ethanol before microneedle application.

Immunization was initiated on day 0 by applying PVA20%-PCL-MNs containing either 10 µg or 100 µg of DNP-KLH to the dorsal skin using manual pressure. The devices were secured in place with medical adhesive tape for at least 1 h. A booster dose was administered on day 30 following the same procedure.

Mice treated with PVA20%-PCL-MNs containing PBS were used as the negative control, whereas mice immunized intraperitoneally with 100 µg of DNP-KLH were used as the positive control. Blood samples were collected from the submandibular vein on days 7, 14, 21, and 28 after the first immunization and at the same time intervals after the booster dose. To obtain serum, blood samples were incubated at 37 °C for 30 min to accelerate clotting and then at 4 °C for an additional 30 min to enhance clot retraction. Serum was recovered by centrifugation at 3381× *g* for 15 min at 4 °C and stored until analysis.

### 2.10. Evaluation of Antigen-Specific IgG Serum Levels by ELISA

DNP-specific antibody titers were determined by ELISA. Briefly, 96-well plates (Nunc-Immuno Maxisorp) were coated with 100 µL per well of dinitrophenyl-conjugated bovine serum albumin (DNP-BSA; Sigma-Aldrich, St. Louis, MO, USA; cat. 324101) at 5 µg mL^−1^ in PBS, pH 7.4, and incubated overnight at 4 °C. Plates were washed three times with PBS containing 0.05% Tween 20 (PBS-T) and blocked with PBS-T containing 10% skimmed milk for 2 h at 37 °C.

After blocking, plates were washed again, and serially diluted serum samples prepared in PBS-T containing 1% skimmed milk were added and incubated overnight at 4 °C. Plates were then washed and incubated with horseradish peroxidase-conjugated anti-mouse IgG antibody (Sigma-Aldrich, St. Louis, MO, USA; cat. A9044) for 1 h at 37 °C.

Reactivity was detected by adding 1 mg mL^−1^ o-phenylenediamine dihydrochloride (OPD; Sigma-Aldrich, St. Louis, MO, USA; cat. P8287) containing 1 µL mL^−1^ H_2_O_2_. After 15 min, stop the reaction by adding 50 µL of 2 M H_2_SO_4_, and measure absorbance at 492 nm using a Multiskan FC microplate reader (Thermo Fisher Scientific, Helsinki, Finland).

The antibody titer was defined as the reciprocal of the highest serum dilution that produced an absorbance value above 0.1 at 492 nm. Blank controls without serum were included in all experiments. Antigen-specific IgG1 and IgG2a responses were determined using the same procedure, with anti-mouse IgG1 (SouthernBiotech, Birmingham, AL, USA; cat. 1071-01) and anti-mouse IgG2a (SouthernBiotech, Birmingham, AL, USA; cat. 1080-01) antibodies, respectively.

### 2.11. Cytokine Quantification

At the end of the immunization protocol, spleens were collected from immunized mice and mechanically dissociated to obtain splenocyte suspensions. Splenocytes were seeded in 24-well plates at a density of 1 × 10^6^ cells per well and stimulated in duplicate with 5 µg mL^−1^ DNP-KLH or DNP-BSA. Plates were incubated for 48 h at 37 °C in a 5% CO_2_ atmosphere. Concanavalin A (ConA)-stimulated cells were used as the positive stimulation control, whereas non-stimulated cells maintained in RPMI medium alone were used as the negative control.

After incubation, plates were centrifuged at 300× *g* for 10 min, and the resulting supernatants were collected and stored at −80 °C until analysis. IL-10 and IFN-γ levels were quantified by ELISA according to the manufacturer’s instructions (BioLegend, San Diego, CA, USA).

Briefly, capture antibodies were immobilized onto ELISA plates for 16 h at room temperature. Plates were washed and blocked for 1 h. Cytokine standards were serially diluted to generate calibration curves. Subsequently, 100 µL of each supernatant was added to the wells and incubated for 2 h at room temperature under shaking. Then, detection antibodies were added and incubated for 2 h. After washing, substrate solution was added to each well and incubated at room temperature for 10 min. Absorbance was measured at 450 nm, and cytokine concentrations were calculated by interpolation from the corresponding standard curves.

### 2.12. Ethics Statement

This study followed the guidelines of the Brazilian National Council for the Control of Animal Experimentation (CONCEA), which follows international guidelines for animal welfare and the principles of the 3Rs. The Ethics Committee on Animal Use of the Butantan Institute, São Paulo, Brazil, approved the experimental protocols involving mice (protocol no. 3752310325), and the study complied with the ARRIVE guidelines. The use of porcine ear skin for ex vivo permeation and insertion assays was submitted to the National Council for Control of Animal Experimentation (CONCEA). The animal study protocol was approved by the Ethics Committee of the Federal University of Sao Paulo (protocol number 1686050526, 25 May 2026).

Mice were housed in a BSL-1 animal facility in microisolators under controlled ventilation, temperature, and light cycle. Animals received food and water ad libitum, and all procedures were performed by trained personnel.

## 3. Results

### 3.1. Ex Vivo Permeation of DNP-KLH from Coated PCL-MNs Across Porcine Ear Skin

The ex vivo permeation of DNP-KLH from coated PCL-MNs was evaluated using porcine ear skin mounted in Franz diffusion cells. This assay was performed to assess the ability of PVA20%-PCL-MNs and ALG4%-PCL-MNs to promote the transport of a macromolecular antigen model across a biologically relevant skin barrier.

As shown in Figure 1, both formulations showed a gradual increase in the absorbance-based estimate of DNP-KLH-equivalent permeation over time. For both PVA20%-PCL-MNs and ALG4%-PCL-MNs, the highest permeated amount was observed after 30 h of experimentation. The permeation profiles showed similar trends, with calculated flux values of 0.30517 ± 0.02 µg cm^−2^ h^−1^ for PVA20%-PCL-MNs and 0.26384 ± 0.02 µg cm^−2^ h^−1^ for ALG4%-PCL-MNs.

These results suggest that both hydrophilic coatings supported DNP-KLH-equivalent transport across porcine skin under the tested conditions, although definitive antigen-specific quantification would require more selective methods. The similarity between the permeation profiles suggests that, once microneedle-mediated skin disruption is achieved, the formation of transient microchannels may play a major role in antigen transport through the skin. Therefore, PVA20%-PCL-MNs and ALG4%-PCL-MNs both promoted ex vivo permeation of the macromolecular antigen model, despite their different coating compositions.

### 3.2. Skin Penetration and Insertion Efficiency of Coated PCL-MNs in Porcine Skin

OCT and optical microscopy analyses were performed to evaluate whether polymer coating affected the insertion performance of PCL-MNs in porcine skin. Porcine skin was selected as a more biologically relevant ex vivo model for assessing microneedle insertion and skin surface disruption. Representative OCT cross-sectional images showed a continuous surface profile for intact porcine skin, whereas skin treated with uncoated PCL-MNs, ALG4%-PCL-MNs, and PVA20%-PCL-MNs exhibited localized surface alterations and indentations consistent with microneedle insertion sites (Figure 2).

Optical microscopy images of porcine skin after microneedle application further confirmed the formation of visible puncture marks for all formulations (Figure 3A). Insertion efficiency was calculated as the percentage of visible insertion sites relative to the total number of microneedles in each array. Uncoated PCL-MNs showed an insertion efficiency of 85%, whereas ALG4%-PCL-MNs and PVA20%-PCL-MNs showed insertion efficiencies of 83% and 81%, respectively (Figure 3B). These results indicate that the coated microneedles maintained good insertion performance after application to porcine skin, with all formulations achieving insertion efficiencies above 80%.

Although uncoated PCL-MNs showed the highest insertion efficiency, the differences among the groups were small. The slight reduction observed for the coated formulations may be associated with changes in tip sharpness, surface geometry, or coating thickness introduced during the coating process. Nevertheless, both ALG4%-PCL-MNs and PVA20%-PCL-MNs largely preserved the ability of PCL-MNs to insert into porcine skin. These findings support the use of coated PCL-MNs as polymeric microdevices for transdermal antigen delivery and provide a more physiologically relevant insertion assessment than standardized synthetic models alone. Complementary Parafilm^®^ insertion assays were also performed as a standardized multilayer model and are provided in the Supporting Information (Figure S1).

### 3.3. Morphological Characterization of Uncoated and Polymer-Coated PCL-MNs by SEM

SEM analysis was performed to further evaluate the effect of polymer coating on the surface morphology and tip geometry of PCL-MNs. Uncoated PCL-MNs showed a well-defined microneedle architecture, with preserved pyramidal geometry and relatively sharp tips (Figure 4A). PVA20%-PCL-MNs exhibited a more evident coating layer associated with partial rounding of the microneedle tips (Figure 4B). In contrast, ALG4%-PCL-MNs showed a less pronounced surface coating, with better preservation of the microneedle geometry and tip definition (Figure 4C).

These morphological differences help explain the insertion behavior observed in porcine skin. Although all formulations produced visible insertion sites, the slight reduction in insertion efficiency observed for the coated microneedles may be related to changes in tip sharpness, surface geometry, or coating thickness introduced during the coating process. In particular, the more evident PVA coating may partially reduce tip sharpness, whereas the alginate coating appears to better preserve the microneedle architecture. Nevertheless, both coated formulations maintained insertion efficiencies above 80% and supported DNP-KLH permeation across porcine skin, indicating that polymer coating did not prevent functional skin insertion or antigen transport under the tested conditions.

### 3.4. Mechanical Compression Behavior of Uncoated and Polymer-Coated PCL-MNs

To further evaluate whether polymer coating affected the mechanical robustness of the microneedle arrays, axial compression tests were performed for uncoated PCL-MNs, PVA20%-PCL-MNs, and ALG4%-PCL-MNs. As shown in Figure 5, all formulations exhibited a nonlinear increase in compression force as a function of displacement, without an abrupt force drop indicative of catastrophic structural failure within the investigated range.

The mechanical response differed among the formulations. Uncoated PCL-MNs reached a maximum compression force of approximately 47 N at a displacement close to 0.89 mm. In contrast, PVA20%-PCL-MNs reached approximately 49 N at a lower displacement, around 0.60 mm, whereas ALG4%-PCL-MNs reached approximately 49 N at approximately 0.70 mm. Thus, although all formulations withstood comparable maximum compression forces, the coated microneedles reached these forces at lower displacement values, indicating greater resistance to axial deformation after polymer coating.

The displacement required to reach 30 N, corresponding to the approximate force applied during the porcine skin insertion assay, was also lower for the coated formulations than for uncoated PCL-MNs. This behavior suggests that PVA20%-PCL-MNs and ALG4%-PCL-MNs maintained sufficient mechanical resistance under compression and may display increased apparent stiffness compared with uncoated PCL-MNs. Importantly, PVA20%-PCL-MNs withstood forces above the insertion force used in the ex vivo porcine skin assay, supporting the conclusion that the partial rounding of the tips observed by SEM did not prevent functional skin insertion under the tested conditions. Together with the OCT and optical microscopy insertion results, these mechanical data indicate that polymer coating did not compromise the structural robustness of the PCL-MN arrays. Instead, PVA and alginate coatings modified the force–displacement response, with coated microneedles showing higher resistance to deformation at lower displacement values.

### 3.5. Biocompatibility Evaluation of PVA20%-PCL-MNs

Cell viability assays were performed using HEK293T epithelial cells as an initial assessment of the in vitro cytocompatibility of PVA20%-PCL-MNs after 24 h of incubation. Cell viability was determined relative to untreated cells, which were considered 100% viable. Ethanol-treated cells were used as a cytotoxic control.

As shown in Figure 6, cells exposed to PVA20%-PCL-MNs maintained high viability when compared with untreated control cells. In contrast, ethanol-treated cells showed a marked reduction in cell viability, confirming the sensitivity of the assay. Although some variation was observed among independent experiments, exposure to PVA20%-PCL-MNs did not markedly reduce HEK293T cell viability after 24 h under the tested conditions.

Although HEK293T cells provided a reliable preliminary model to confirm the general non-cytotoxic nature of the PVA20%-PCL formulation, further evaluation using skin-specific models remains crucial to fully validate cutaneous biocompatibility for transdermal delivery. Nevertheless, these results demonstrate preliminary in vitro cytocompatibility of PVA20%-PCL-MNs, supporting their further evaluation in animal experiments as a proof-of-concept transdermal delivery platform.

### 3.6. Evaluating DNP-KLH Integrity and Stability Post-Release from MNs

The integrity and stability of DNP-KLH released from the PVA20%-PCL MNs were evaluated by SDS-PAGE analysis (Figure S2). Free DNP-KLH (4 µg) was loaded as a control. Due to the high molecular weight and complex oligomeric structure of KLH, the protein species were predominantly retained at the top of the resolving gel across all tested conditions. Despite this limited migration, intact DNP-KLH was clearly visible in all released MN formulations tested. A clear concentration-dependent intensity was observed, MNs loaded with 100 µg of antigen showed noticeably higher band intensity compared to the 10 µg MN group, indicating a greater amount of released protein after 1 h of incubation in DPBS. Overall, the presence of the characteristic high-molecular-weight protein band confirms that DNP-KLH remained intact during MN fabrication and subsequent release, without significant degradation into lower-molecular-weight fragments.

### 3.7. Humoral Immune Response Induced by PVA20%-PCL-MNs Containing DNP-KLH

To evaluate whether PVA20%-PCL-MNs could induce an antigen-specific humoral immune response, BALB/c mice were immunized transdermally with two doses of PVA20%-PCL-MNs containing either 10 µg or 100 µg of DNP-KLH. Mice treated with PVA20%-PCL-MNs containing PBS were used as the negative control, whereas mice immunized intraperitoneally with 100 µg of DNP-KLH were used as the positive control. Intraperitoneal administration was selected as a standardized positive control to establish the maximum hapten-specific IgG production. Blood samples were collected on days 7, 14, 21, and 28 after the first immunization. A booster dose was administered on day 30, followed by additional blood collections at the same time intervals. DNP-specific IgG titers were evaluated by ELISA.

After the first immunization, mice treated with PVA20%-PCL-MNs containing either 10 µg or 100 µg of DNP-KLH showed modest DNP-specific IgG responses compared with the negative control group (Figure 7A). The intraperitoneally immunized positive control group showed higher IgG titers from day 7 after the first dose, which remained elevated throughout the analysis. Following the booster dose, IgG titers increased in both MN-immunized groups, with a more pronounced response in animals receiving 100 µg of DNP-KLH (Figure 7B). Although the antibody titers induced by transdermal immunization were lower than those observed after intraperitoneal immunization, the booster-dependent increase supports the ability of PVA20%-PCL-MNs to deliver DNP-KLH and stimulate an antigen-specific humoral response.

These results indicate that PVA20%-PCL-MNs can promote antigen-specific IgG responses after transdermal administration of DNP-KLH, particularly after booster immunization. Therefore, although the magnitude of the response was lower than that induced by intraperitoneal immunization, the data support the feasibility of this polymeric microneedle platform as a proof-of-concept system for transdermal antigen delivery.

### 3.8. IgG2a/IgG1 Ratio in Mice Immunized with PVA20%-PCL-MNs Containing DNP-KLH

To further investigate the humoral immune profile induced by PVA20%-PCL-MNs containing DNP-KLH, the antigen-specific IgG2a/IgG1 ratio was evaluated as an indicator of Th1- and Th2-associated responses. Serum samples from each group were pooled and analyzed in triplicate. The ratios were calculated by dividing the IgG2a absorbance values by the corresponding IgG1 absorbance values. Ratios higher than 1.0 are generally associated with a Th1-skewed response, whereas ratios lower than 1.0 are associated with a Th2-skewed response.

As shown in Figure 8, all groups and time points showed IgG2a/IgG1 ratios below 1.0, indicating a predominantly Th2-associated humoral profile. After the first immunization, mice treated with PVA20%-PCL-MNs containing either 10 µg or 100 µg of DNP-KLH showed low IgG2a/IgG1 ratios on days 7 and 14, similar to those observed in the negative control group (Figure 8A). By day 21, an increase in the IgG2a/IgG1 ratio was observed in the PVA20%-PCL-MN-treated groups, particularly in animals receiving 100 µg of DNP-KLH, reaching values closer to those observed in the intraperitoneally immunized positive control group. However, the ratios remained below 1.0, indicating maintenance of a Th2-associated profile.

After the booster dose, both MN-immunized groups showed sustained IgG2a/IgG1 ratios below 1.0 throughout the analyzed period (Figure 8B). The 100 µg DNP-KLH MN group showed higher ratios than the 10 µg group, especially at later time points, suggesting a dose-dependent increase in IgG2a contribution after booster immunization. Nevertheless, the overall profile remained predominantly IgG1-biased. The intraperitoneally immunized positive control group also showed IgG2a/IgG1 ratios below 1.0, consistent with a Th2-associated humoral response.

These results suggest that transdermal immunization with PVA20%-PCL-MNs containing DNP-KLH induced a predominantly Th2-associated antibody profile, with detectable IgG2a contribution after booster immunization, particularly at the higher antigen dose. Because pooled serum samples were used, these data should be interpreted as group-level trends rather than as evidence of statistically significant differences among individual animals.

### 3.9. Cytokine Production by Splenocytes from Mice Immunized with PVA20%-PCL-MNs Containing DNP-KLH

At the end of the immunization protocol, spleens were collected from immunized mice, mechanically dissociated, and splenocytes were cultured in RPMI medium. Cells from all experimental groups were stimulated in vitro with DNP-KLH or DNP-BSA. Non-stimulated cells served as the negative control, whereas ConA-stimulated cells were used as the positive stimulation control. The levels of IFN-γ and IL-10 in the culture supernatants were quantified by ELISA.

As shown in Figure 9A, splenocytes from mice immunized with PVA20%-PCL-MNs containing 10 µg of DNP-KLH produced increased levels of IFN-γ after stimulation with DNP-KLH. A lower but still detectable IFN-γ response was observed in the group immunized with PVA20%-PCL-MNs containing 100 µg of DNP-KLH. In contrast, splenocytes from intraperitoneally immunized mice showed low IFN-γ production under the same stimulation conditions.

IL-10 production was also detected after DNP-KLH stimulation, particularly in splenocytes from mice immunized with PVA20%-PCL-MNs containing either 10 µg or 100 µg of DNP-KLH (Figure 9B). The intraperitoneally immunized group also produced IL-10 upon recall stimulation. Stimulation with DNP-BSA induced lower cytokine levels than DNP-KLH across MN-immunized groups, reinforcing the antigen specificity of the recall response toward the hapten-carrier complex used for immunization.

These results indicate that transdermal immunization with PVA20%-PCL-MNs loaded with DNP-KLH primed antigen-specific splenocytes capable of cytokine secretion upon restimulation. The concomitant detection of IFN-γ and IL-10 suggests a mixed cytokine response elicited by the MNs. However, as these measurements were performed in bulk splenocyte cultures evaluating a limited cytokine panel, these findings demonstrate functional antigen-specific immune activation rather than definitive CD4+ T-cell subset polarization (Th1/Th2).

## 4. Discussion

Vaccination remains one of the most effective public health interventions for preventing infectious diseases. However, conventional needle-and-syringe administration still presents practical limitations, including pain, needle phobia, the need for trained personnel, and challenges associated with vaccine distribution and storage. These limitations have motivated the development of alternative delivery technologies that simplify administration and improve patient acceptance. In this context, microneedle-based platforms have emerged as promising polymeric microdevices for minimally invasive transdermal delivery, as they can access the epidermal and dermal compartments, which are rich in antigen-presenting cells [20,21,22].

In the present study, PCL-based microneedles coated with hydrophilic polymers were evaluated as a proof-of-concept platform for transdermal antigen delivery. PCL was selected as the structural polymer due to its biocompatibility, processability, and prior use in biomedical devices. In this system, PCL provides the mechanical support required for microneedle fabrication, whereas the hydrophilic polymeric coating acts as a functional interface for antigen loading and release. This design is particularly relevant for polymeric microdevice applications, as it separates the structural role of the microneedle substrate from the release function provided by the coating layer.

The ex vivo permeation assay using porcine ear skin provided a biologically relevant assessment of DNP-KLH transport across the skin after microneedle application. This represents an important improvement over assays based only on low-molecular-weight model probes, since DNP-KLH is a macromolecular antigen and its transport through the skin is expected to be influenced by molecular size, coating hydration, antigen diffusion, and the transient microchannels generated by microneedle insertion. Both PVA20%-PCL-MNs and ALG4%-PCL-MNs promoted gradual DNP-KLH permeation over 30 h, with similar permeation profiles and comparable flux values. These findings suggest that, under the tested ex vivo conditions, the ability of the microneedles to disrupt the skin barrier and create transport pathways may have a stronger influence on antigen permeation than the specific hydrophilic coating composition.

This result is particularly relevant because PVA and alginate coatings showed distinct morphological features. SEM analysis indicated that PVA20%-PCL-MNs formed a more evident coating layer associated with partial rounding of the microneedle tips, whereas ALG4%-PCL-MNs better preserved the microneedle geometry. Such morphological differences could affect insertion performance by modifying tip sharpness, surface geometry, or force transfer during application. Nevertheless, OCT and optical microscopy analyses in porcine skin confirmed that uncoated PCL-MNs, ALG4%-PCL-MNs, and PVA20%-PCL-MNs produced detectable insertion sites. The insertion efficiencies observed for all formulations were above 80%, indicating that both coatings largely preserved the ability of the PCL arrays to penetrate porcine skin.

The mechanical compression tests further supported this interpretation. All formulations exhibited a progressive increase in compression force as a function of displacement, without an abrupt force drop indicative of catastrophic structural failure within the investigated range. Although the force–displacement profiles were not identical, PVA20%-PCL-MNs and ALG4%-PCL-MNs reached high compression forces at lower displacement values than uncoated PCL-MNs, indicating increased resistance to axial deformation after polymer coating. Importantly, all formulations withstood forces above 30 N, which corresponds to the approximate force applied during the porcine skin insertion assay. Therefore, despite the partial rounding of the microneedle tips observed for PVA20%-PCL-MNs, the combined mechanical compression, OCT, and optical microscopy results support that polymer-coated PCL-MNs maintained sufficient mechanical robustness for functional skin insertion under the tested conditions.

The similarity between the PVA20%-PCL-MN and ALG4%-PCL-MN permeation profiles helps refine the interpretation of coating performance. Rather than indicating that one coating is universally superior, the results suggest that each coating provides a different balance between morphology, insertion behavior, and antigen transport. Alginate better preserved microneedle geometry, whereas PVA showed more evident coating deposition and partial tip rounding. However, this morphological alteration was not sufficient to prevent insertion into porcine skin or DNP-KLH permeation across the tissue. Complementary Parafilm^®^ assays, presented in the Supporting Information, further confirmed formulation-dependent differences in insertion behavior under standardized multilayer conditions, but the main interpretation of skin insertion in this revised version is supported by the more biologically relevant porcine skin model.

It is important to note that the permeation assay estimates DNP-KLH transport based on absorbance measurements and should be interpreted as DNP-KLH-equivalent permeation. Although this approach provides useful quantitative information on antigen transport across porcine skin, it does not directly demonstrate antigen integrity, conformational preservation, or immunoreactivity after permeation. In addition, because absorbance at 280 nm is not antigen-specific, possible skin-derived components released into the receptor medium during the 30 h Franz diffusion assay may interfere with the measurement. Future studies using blank skin controls, antigen-specific ELISA, protein integrity assays, or immunoreactivity-based detection would help establish the relationship between permeated antigen, structural preservation, and immunogenic activity.

The in vitro cytocompatibility assay further supported the use of PVA20%-PCL-MNs in subsequent animal experiments. HEK293T cells exposed to the microneedles maintained high viability after 24 h of incubation, whereas ethanol-treated cells showed a marked decrease in viability. These results indicate that PVA20%-PCL-MNs showed preliminary cytocompatibility under the tested conditions. However, it is important to note that the MTT assay evaluates cell metabolic activity and does not directly demonstrate sterility, long-term biocompatibility, or local skin safety. Additional studies using skin-relevant cell models, such as keratinocytes or fibroblasts, as well as extended culture periods and microbiological assays, would provide complementary information regarding the safety profile of this platform.

Transdermal immunization using PVA20%-PCL MNs loaded with DNP-KLH induced detectable antigen-specific IgG responses in mice, with a pronounced enhancement following booster administration. While the antibody titers elicited by the MNs were lower than those achieved by the intraperitoneal positive control, a standardized benchmark used to induce maximal humoral responses, the increase in booster-dependent IgG demonstrates that the MN system delivered functional antigen across the cutaneous barrier in amounts sufficient to prime and boost systemic humoral immunity. These results corroborate PVA-coated PCL microneedles as a viable polymeric platform for transdermal vaccine delivery.

The analysis of the IgG2a/IgG1 ratio provided further insight into the humoral immune profile induced by the platform. The ratios remained below 1.0 across groups and time points, indicating a predominantly Th2-associated profile. After booster immunization, both MN-immunized groups showed detectable IgG2a contribution, particularly at the higher antigen dose, but the overall response remained IgG1-biased. Because serum samples from each group were pooled and analyzed in technical triplicates, these data should be interpreted as group-level trends rather than as evidence of statistically significant differences among individual animals.

The cytokine profile from restimulated splenocytes confirms that transdermal immunization via PVA20%-PCL-MNs effectively primed systemic cellular immunity. Splenocytes from mice immunized with DNP-KLH-loaded microneedles produced detectable levels of both IFN-γ and IL-10 upon in vitro recall with DNP-KLH, demonstrating antigen-specific reactivity. The concomitant release of IFN-γ and IL-10 points toward a balanced immune response. However, because these evaluations were conducted on bulk splenocyte cultures using a limited cytokine panel, these data primarily demonstrate functional cellular priming rather than definitive CD4+ T-cell subset lineage commitment. To fully delineate the fine T-cell polarization mechanisms and cell populations driving this response, future studies incorporating expanded cytokine profiling and flow cytometry-based immunophenotyping are warranted.

The ability of microneedles to induce immune responses may be linked to their interaction with the skin immune environment. Transdermal delivery can promote antigen deposition in tissues enriched with antigen-presenting cells, including Langerhans cells and dermal dendritic cells. This local immunological context may help explain why microneedle-based immunization can elicit humoral responses and antigen-stimulated cytokine production, even when total antibody titers are lower than those induced by systemic intraperitoneal administration. Previous studies using vaccine-coated microneedles have similarly reported induction of antibody responses and cytokine-producing cells, supporting the broader potential of this delivery route for immunization strategies [23,24,25].

The results demonstrate that PVA20%-PCL-MNs constitute a promising polymeric microneedle platform for transdermal antigen delivery. The system combines a mechanically stable PCL microneedle substrate with a hydrophilic PVA coating that supports antigen loading, skin insertion, and DNP-KLH permeation across porcine skin. Although SEM analysis showed that the PVA coating partially altered tip morphology, OCT and optical microscopy confirmed that this effect did not prevent insertion into porcine skin. In vivo, the platform induced booster-dependent antigen-specific IgG responses and detectable cytokine production after splenocyte stimulation. These findings support using PVA-coated PCL microneedles as functional polymeric microdevices for antigen delivery. At the same time, the study should be viewed as a proof of concept. Future work should focus on directly quantifying antigen loading and delivery efficiency, including antigen distribution between microneedle tips and the backing substrate, as well as the amount of antigen retained in the skin after application. Additional studies should also evaluate coating uniformity and thickness, antigen stability during storage and after permeation, dose-delivery reproducibility, broader skin biocompatibility, and comparisons with clinically relevant immunization routes. Such studies will be essential to establish the full potential of this platform for vaccine delivery applications.

## 5. Conclusions

This study demonstrates the feasibility of using PVA-coated PCL microneedles as a polymeric microdevice platform for transdermal antigen delivery. Ex vivo permeation assays using porcine ear skin provided an absorbance-based estimate of DNP-KLH-equivalent transport across a biologically relevant skin barrier, with PVA20%-PCL-MNs showing a permeation profile comparable to that observed for ALG4%-PCL-MNs. SEM analysis showed that PVA coating partially altered microneedle tip morphology. However, mechanical compression testing, together with OCT and optical microscopy analyses, confirmed that PVA20%-PCL-MNs maintained the ability to insert into porcine skin and generate visible insertion sites. PVA20%-PCL-MNs also maintained preliminary in vitro cytocompatibility in HEK293T cells under the tested conditions. When loaded with DNP-KLH, the PVA20%-PCL-MN platform induced booster-dependent antigen-specific IgG responses in mice and promoted detectable IFN-*γ* and IL-10 production after splenocyte stimulation. Although the immune response induced by microneedles was lower than that observed after intraperitoneal immunization, the results support the potential of this system as a proof-of-concept platform for minimally invasive antigen delivery. Future studies should focus on direct quantification of antigen loading and delivery efficiency, coating uniformity and thickness, antigen stability after permeation and during storage, broader skin biocompatibility assessment, dose-delivery reproducibility, and comparisons with clinically relevant immunization routes.

## Figures and Tables

**Figure 1:**
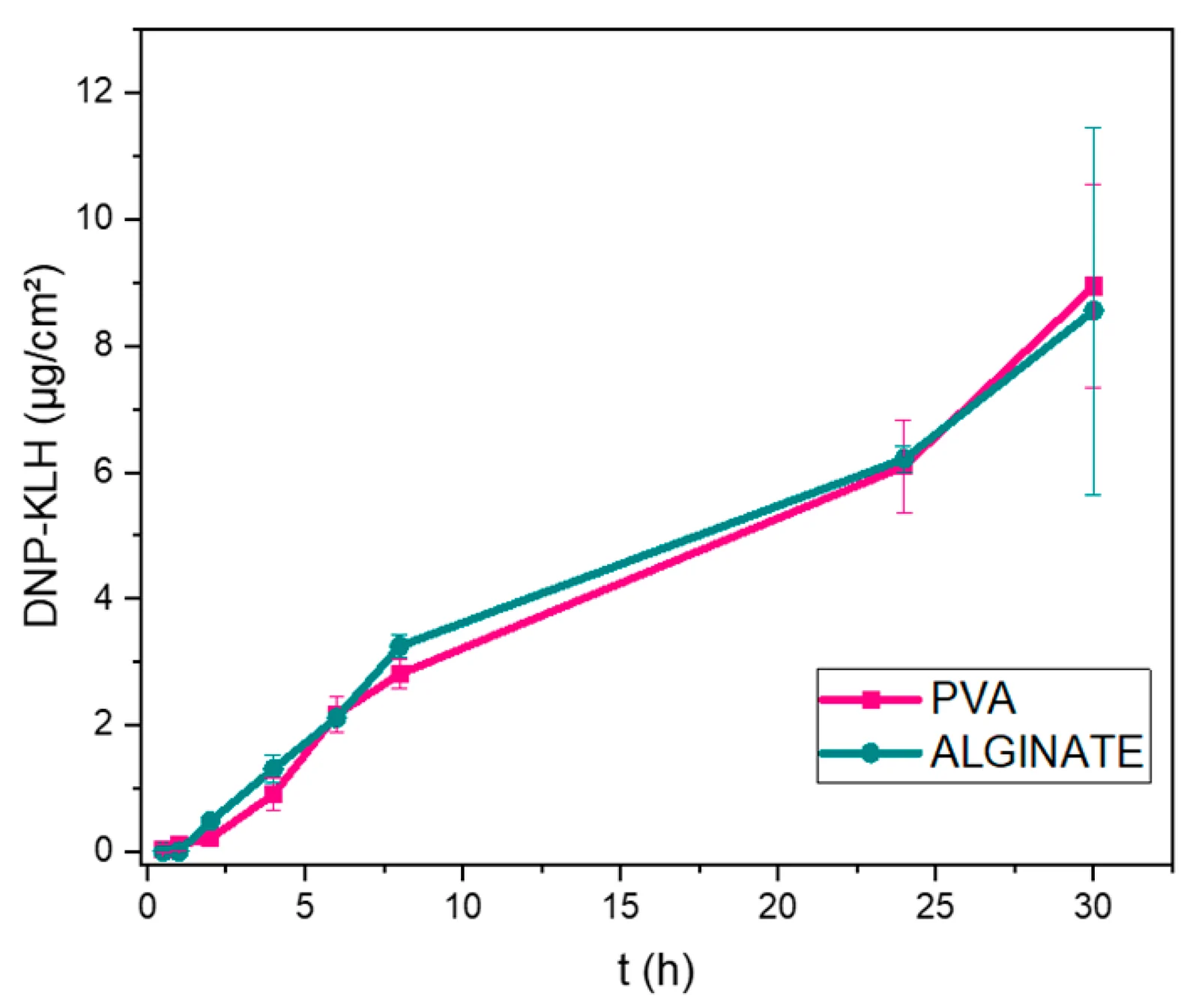
Ex vivo DNP-KLH-equivalent permeation from polymer-coated PCL microneedles. Absorbance-based estimate of DNP-KLH-equivalent permeation from PVA20%-PCL-MNs and ALG4%-PCL-MNs across porcine ear skin using Franz diffusion cells over 30 h. Data are expressed as mean ± SD, with n = 5 microneedle samples for each formulation at each time point.

**Figure 2:**
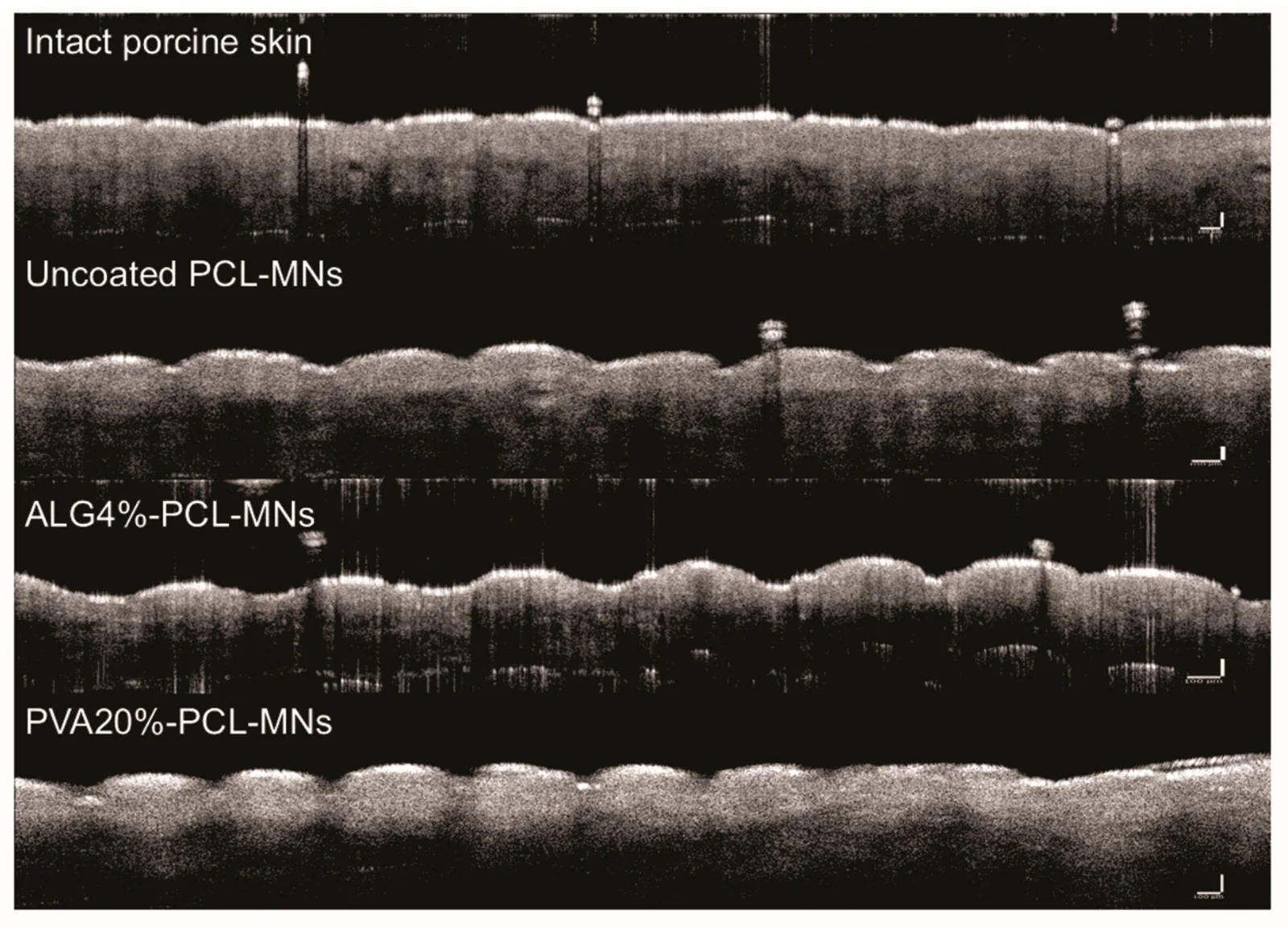
Optical coherence tomography (OCT) assessment of microneedle application to porcine skin. Representative cross-sectional OCT images of intact porcine skin and porcine skin after application of uncoated PCL-MNs, ALG4%-PCL-MNs, and PVA20%-PCL-MNs. Intact skin showed a more continuous surface profile, whereas microneedle-treated samples exhibited localized surface deformation and indentations associated with microneedle application. Scale bars: 100 µm.

**Figure 3:**
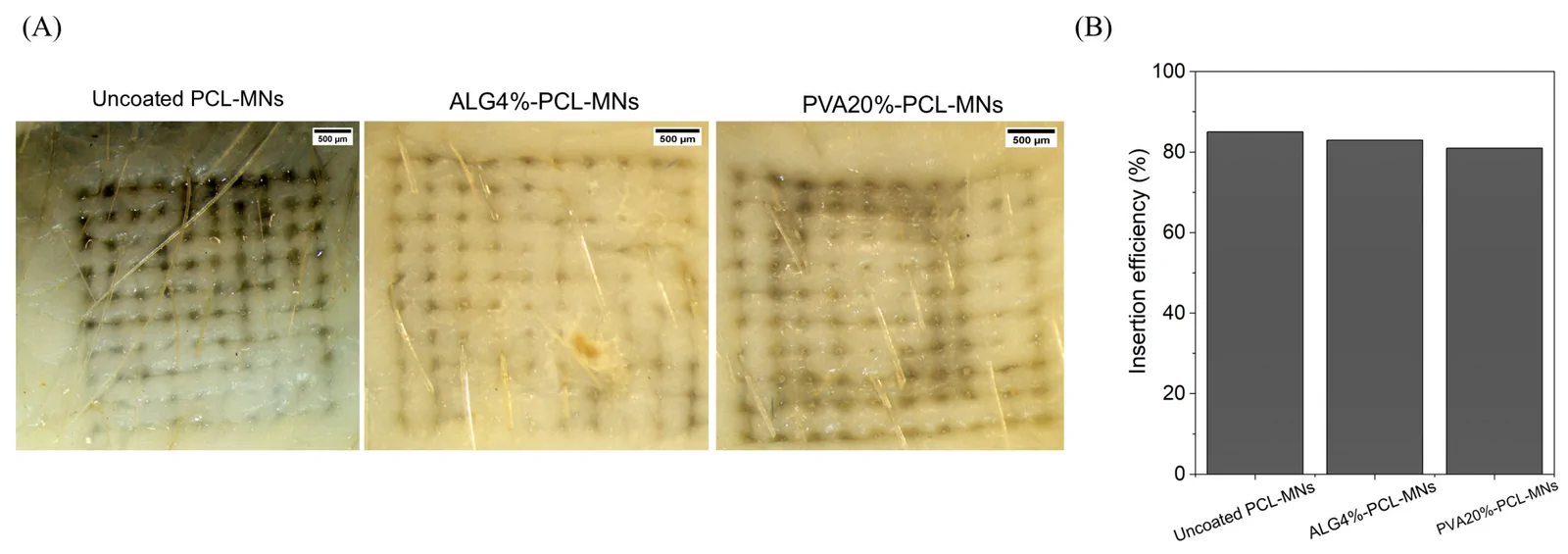
Microneedle insertion sites and insertion efficiency in porcine skin. (**A**) Representative optical microscopy images of porcine skin after application of uncoated PCL-MNs, ALG4%-PCL-MNs, and PVA20%-PCL-MNs. Visible micro-holes distributed within the treated skin area were used to estimate insertion efficiency. Scale bars: 500 µm. (**B**) Insertion efficiency, calculated as the percentage of visible insertion sites relative to the total number of microneedles in each array.

**Figure 4:**
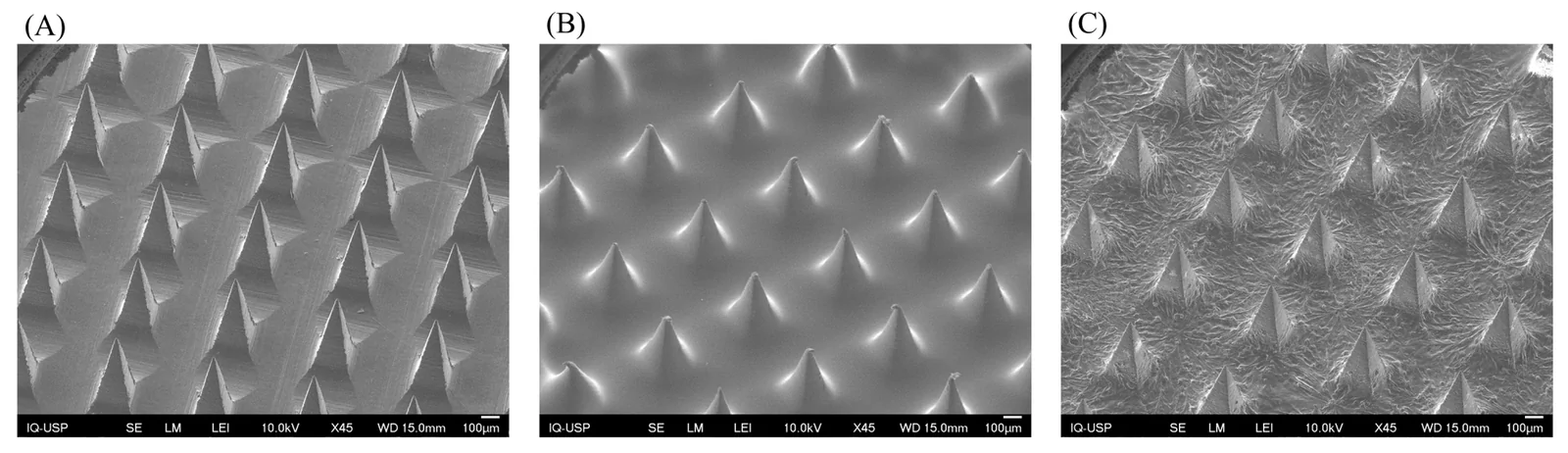
SEM micrographs of uncoated and polymer-coated PCL microneedles. Representative images of uncoated PCL-MNs (**A**), PVA20%-PCL-MNs (**B**), and ALG4%-PCL-MNs (**C**). Uncoated PCL-MNs showed well-defined microneedle geometry, whereas PVA20%-PCL-MNs exhibited a more evident surface coating with partial rounding of the microneedle tips. ALG4%-PCL-MNs showed a less pronounced coating morphology and better preservation of microneedle geometry. Scale bars: 100 µm.

**Figure 5:**
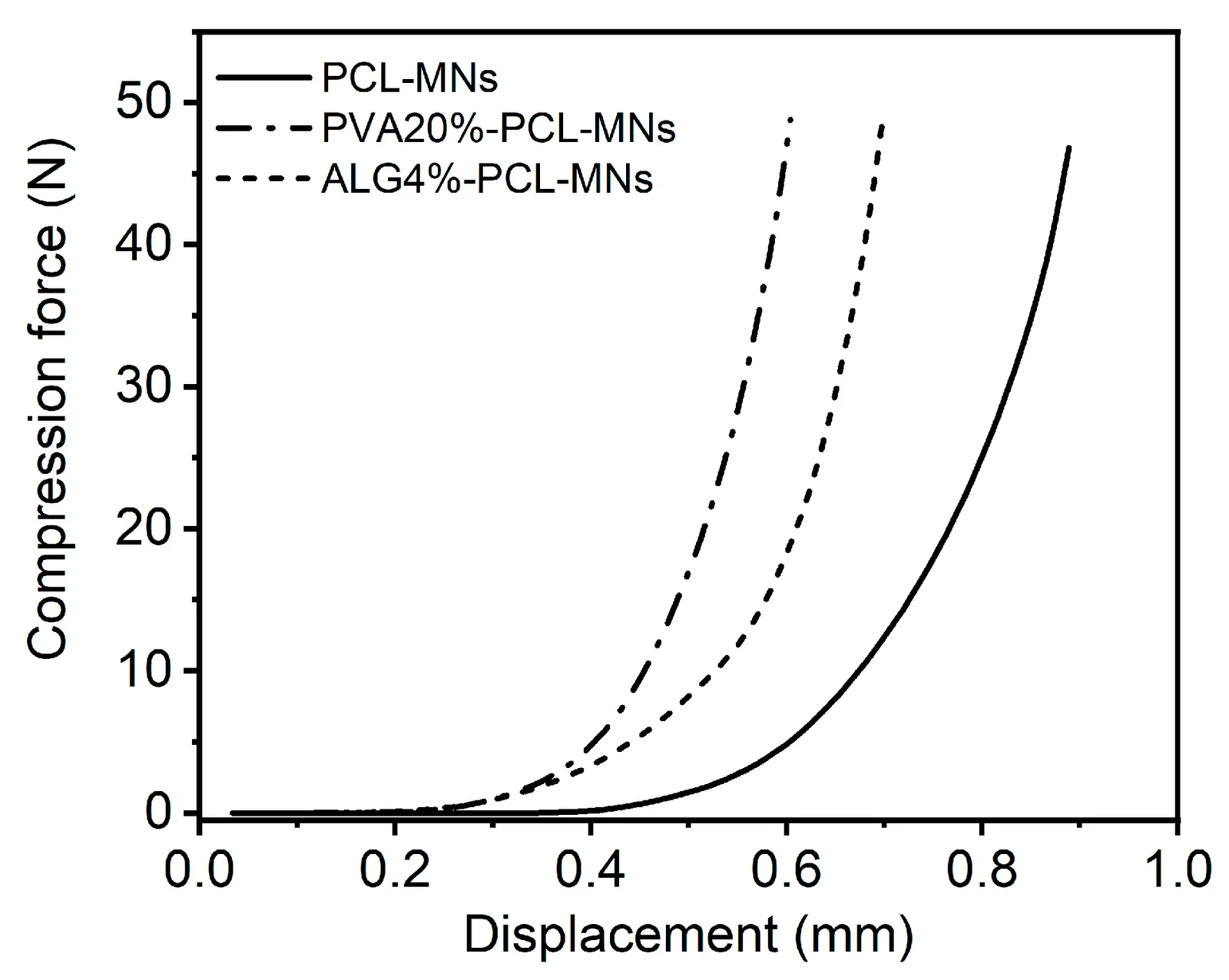
Mechanical compression behavior of uncoated and polymer-coated PCL microneedles. Force–displacement curves obtained for uncoated PCL-MNs, PVA20%-PCL-MNs, and ALG4%-PCL-MNs under axial compression.

**Figure 6:**
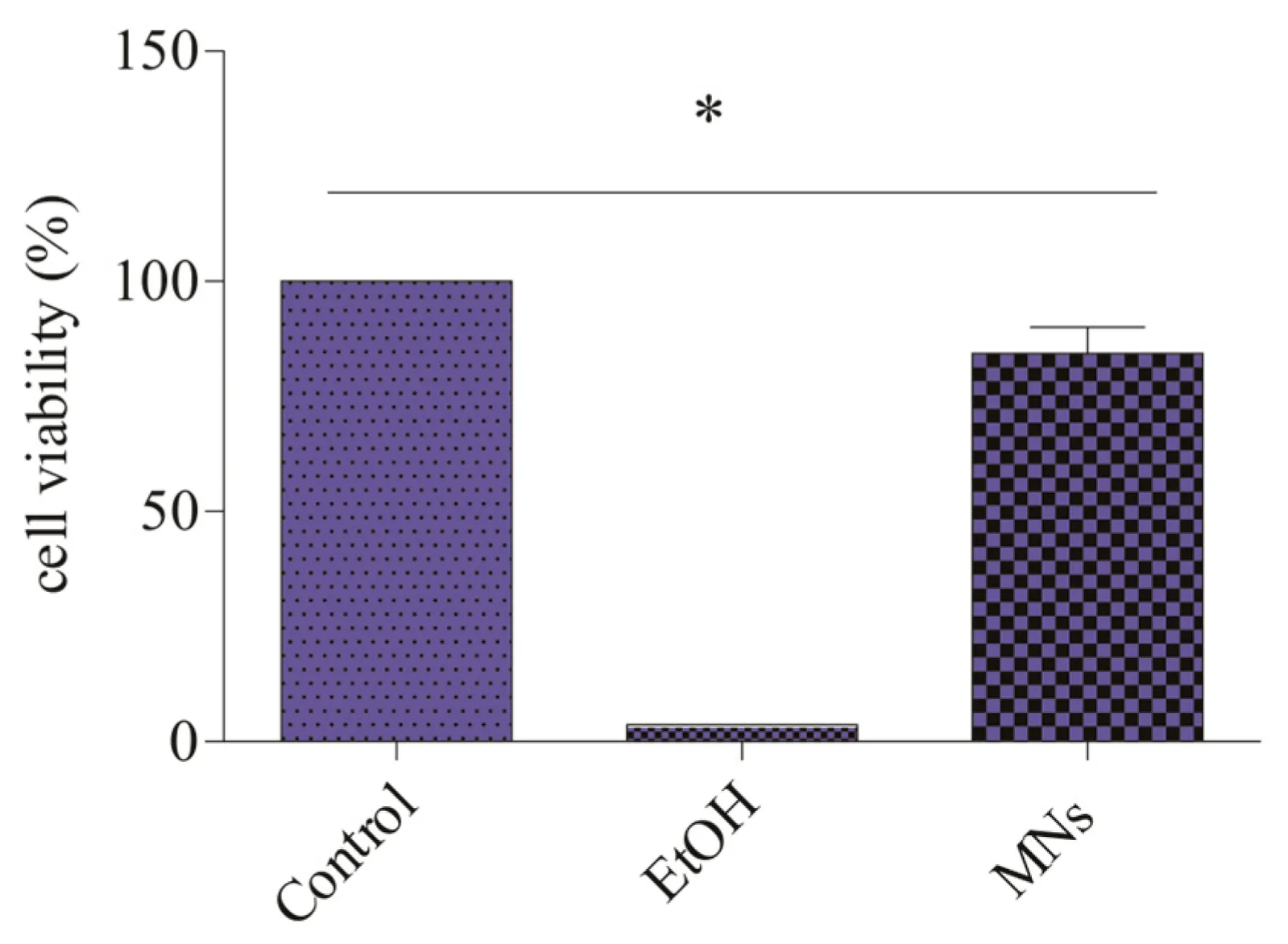
In vitro cell viability of HEK293T cells exposed to PVA20%-PCL-MNs. HEK293T cells were seeded at a density of 1 × 10^4^ cells per well and incubated with PVA20%-PCL-MNs for 24 h. Cell viability was evaluated using the MTT assay, and the percentage of viable cells was calculated relative to untreated control cells, which were considered 100% viable. Ethanol-treated cells were used as a cytotoxic control. Bars represent the mean ± SEM of triplicate measurements from a representative experiment performed three times. Statistical analysis was performed using an unpaired *t*-test; * *p* < 0.05.

**Figure 7:**
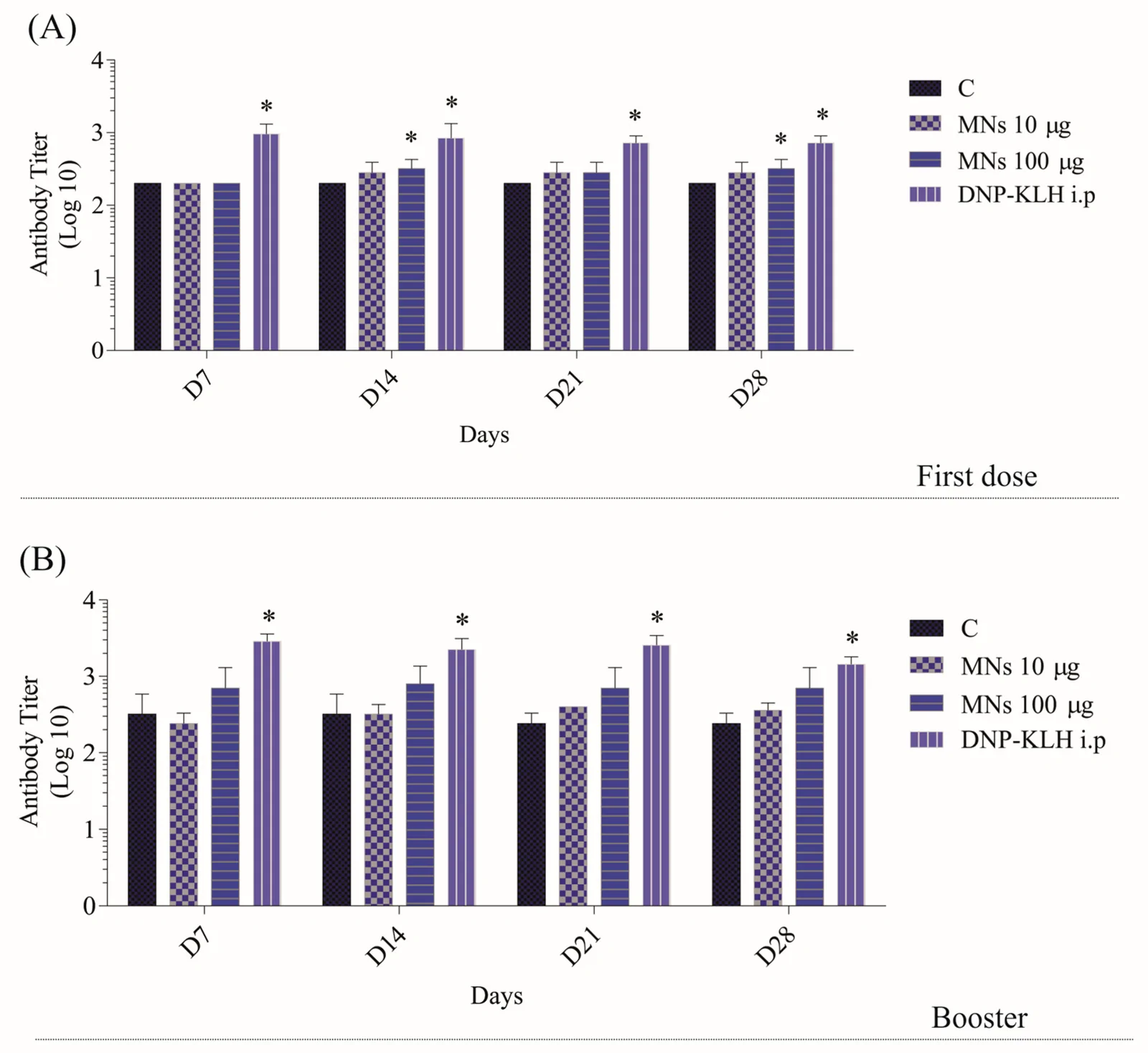
DNP-specific IgG antibody titers in BALB/c mice immunized with PVA20%-PCL-MNs containing DNP-KLH. Mice were immunized transdermally with PVA20%-PCL-MNs containing 10 µg or 100 µg of DNP-KLH. Mice treated with PVA20%-PCL-MNs containing PBS were used as the negative control (C), whereas mice immunized intraperitoneally with 100 µg of DNP-KLH were used as the positive control. Blood samples were collected on days 7, 14, 21, and 28 after the first immunization (**A**). Mice received a booster dose on day 30, and blood collections were performed at the same time intervals after the booster (**B**). Antibody titers are presented as log10 values and expressed as mean ± SD. Statistical analysis was performed using two-way ANOVA with Sidak’s multiple comparisons test. Significant differences relative to the negative control group are denoted by asterisks (*p* < 0.05).

**Figure 8:**
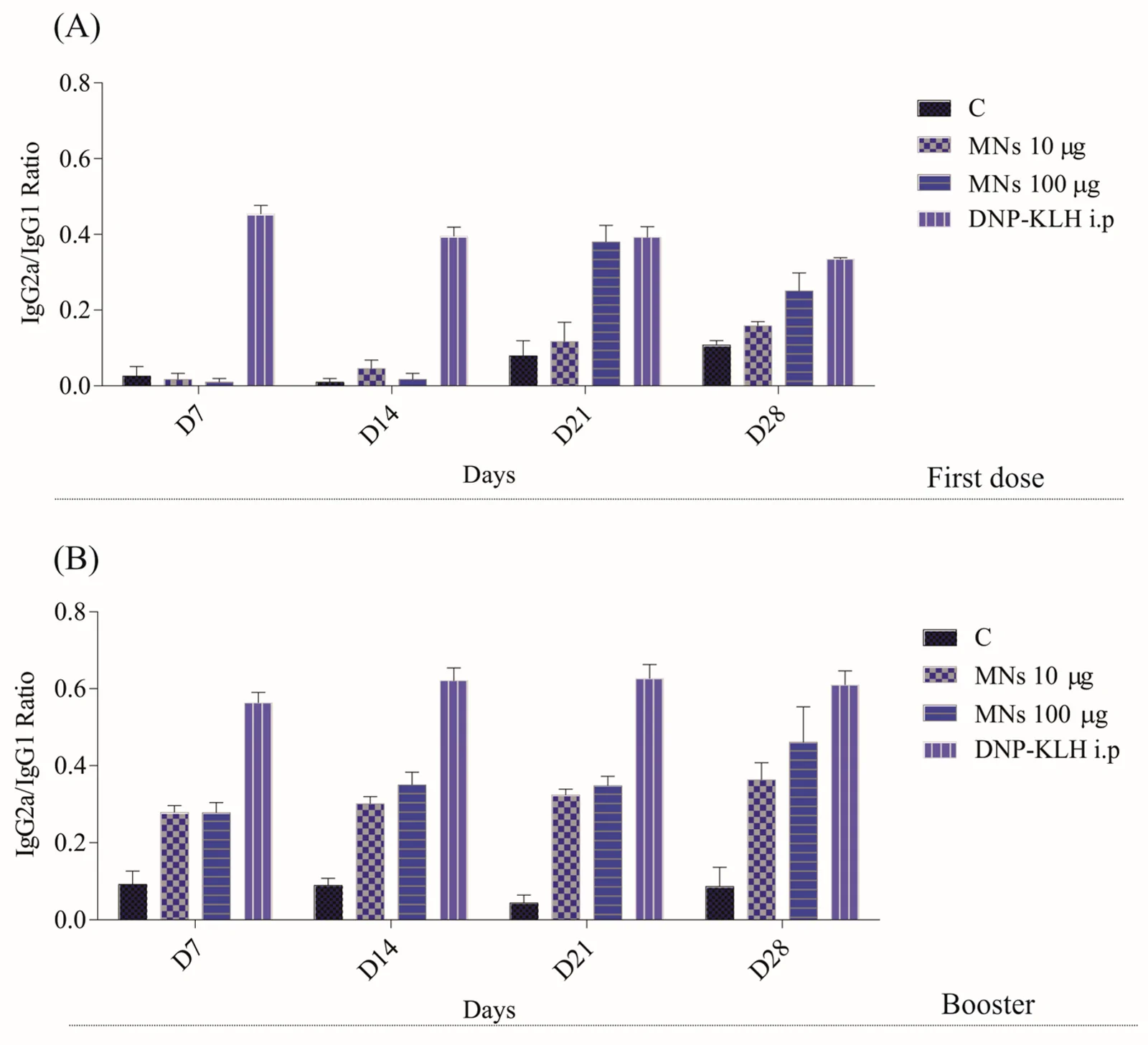
IgG2a/IgG1 ratios of DNP-specific antibodies in BALB/c mice immunized with PVA20%-PCL-MNs containing DNP-KLH. Mice were immunized transdermally with PVA20%-PCL-MNs containing 10 µg or 100 µg of DNP-KLH. Mice treated with PVA20%-PCL-MNs without DNP-KLH were used as the negative control (C), whereas mice immunized intraperitoneally with 100 µg of DNP-KLH were used as the positive control. Blood samples were collected on days 7, 14, 21, and 28 after the first immunization (**A**). Mice received a booster dose on day 30, and blood collections were performed at the same time intervals after the booster (**B**). Serum samples from each group were pooled and analyzed in triplicate. Ratios were calculated by dividing IgG2a absorbance values by the corresponding IgG1 absorbance values. Ratios higher than 1.0 are generally associated with a Th1-skewed response, whereas ratios lower than 1.0 are associated with a Th2-skewed response. Bars represent mean ± SEM of technical triplicates.

**Figure 9:**
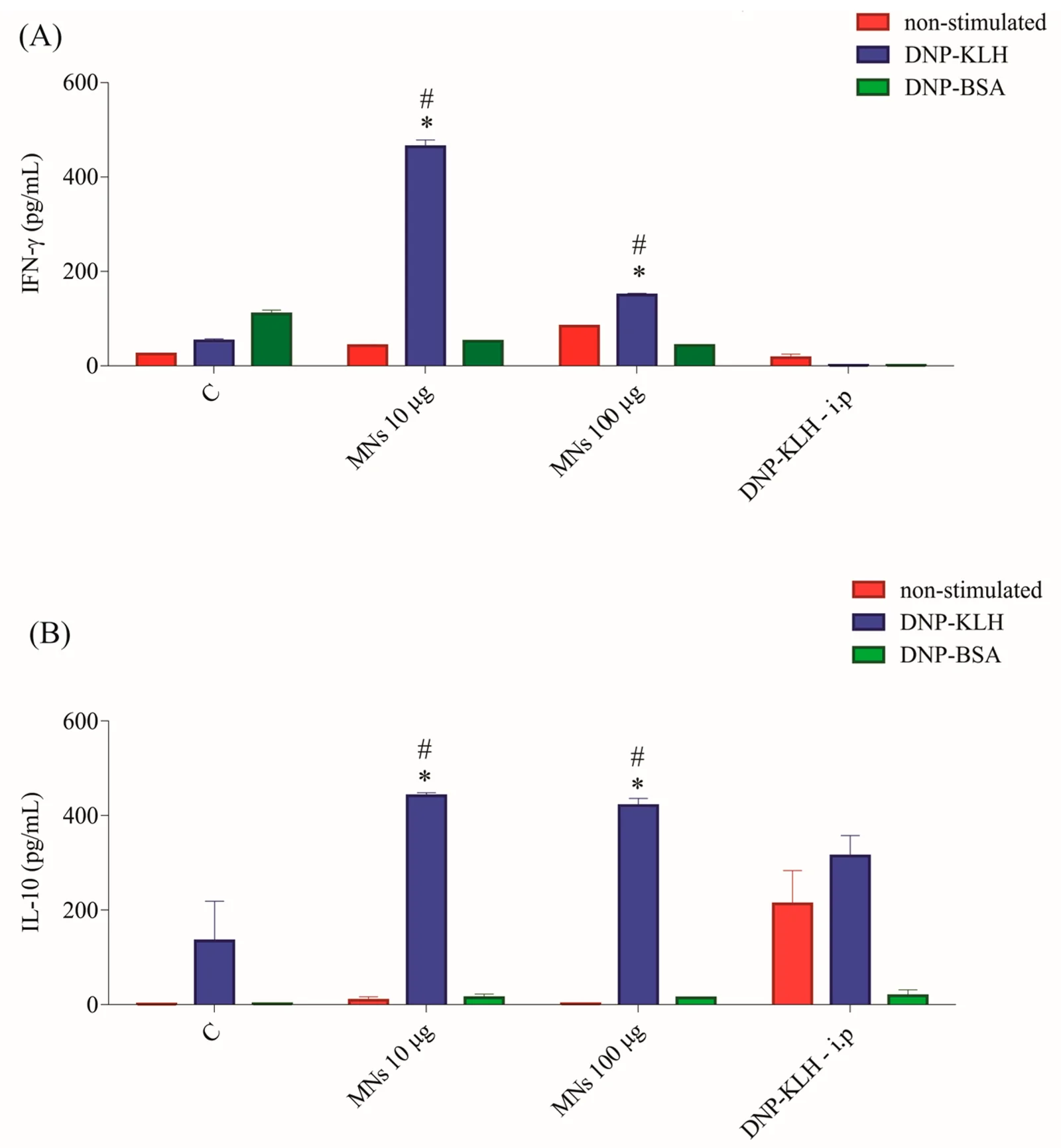
Cytokine production by splenocytes from mice immunized with PVA20%-PCL-MNs containing DNP-KLH. At the end of the immunization protocol, spleens were collected, mechanically dissociated, and splenocytes were cultured at a density of 1 × 10^6^ cells per well. Cells were stimulated in vitro with DNP-KLH or DNP-BSA at 5 µg mL^−1^ for 48 h. Non-stimulated cells were maintained in RPMI medium alone, whereas ConA-stimulated cells were used as the positive stimulation control. IFN-γ (**A**) and IL-10 (**B**) levels in the culture supernatants were quantified by ELISA. Samples were evaluated in duplicate. Data were analyzed by two-way ANOVA with Dunnett’s multiple comparisons test. Asterisks indicate significant differences relative to the corresponding non-stimulated condition, and hash symbols indicate significant differences relative to the negative control group after DNP-KLH stimulation (*p* < 0.05).

## Data Availability

The original contributions presented in this study are included in the article/Supplementary Material. Further inquiries can be directed to the corresponding authors.

## Supplementary Materials

The following supporting information can be downloaded at https://www.mdpi.com/article/10.3390/polym18192327/s1. Figure S1: Parafilm^®^ insertion efficiency of uncoated and polymer-coated PCL-MNs. The percentage of holes per Parafilm^®^ layer was evaluated using an eight-layer Parafilm^®^ model after application of uncoated PCL-MNs, PVA20%-PCL-MNs, and ALG4%-PCL-MNs. Representative images of perforated Parafilm^®^ sheets are shown for each formulation. The assay was performed in triplicate and used as a complementary standardized insertion model; Figure S2: Structural integrity of DNP-KLH released from PVA20%-PCL MNs. SDS-PAGE analysis (4–20% gradient) of DNP-KLH recovered after 1 h release in DPBS 1X from PVA20%-PCL MNs formulations containing 10 µg or 100 µg of DNP-KLH. Lanes: M. molecular mass; 1. DNP-KLH (4 µg) was used as a reference control; 2. empty; 3–4 PVA20%-PCL MNs containing 10 µg of DNP-KLH; 5–6. PVA20%-PCL MNs containing 100 µg of DNP-KLH; 7. empty.
