# Supplementary material for: Polyvinyl Alcohol-Coated Polycaprolactone Microneedles as Polymeric Microdevices for Transdermal Antigen Delivery and Immune Activation
Source: Polymers (Basel). 2026 Sep 24;18(19):2327. doi: 10.3390/polym18192327 (PMC13666606; doi:10.3390/polym18192327)
Supplement: Supplementary file 1 [file polymers-18-02327-s001.zip › polymers-4538664-supplementary.pdf]

Supplementary Parafilm® insertion assay

A Parafilm® insertion assay was performed as a complementary standardized model to compare the perforation performance of uncoated and polymer-coated PCL-MNs. Although this model does not fully reproduce the mechanical properties of biological skin, it provides a reproducible multilayer substrate for comparing the relative insertion behavior of different microneedle formulations. The results showed that insertion efficiency decreased with increasing Parafilm® layer depth for all formulations. Uncoated PCL-MNs showed the highest perforation efficiency, whereas ALG4%-PCL-MNs exhibited an insertion profile closer to that of uncoated PCL-MNs. PVA20%-PCL-MNs showed a more pronounced reduction in perforation efficiency, suggesting that the PVA coating partially affected insertion performance in this standardized model.

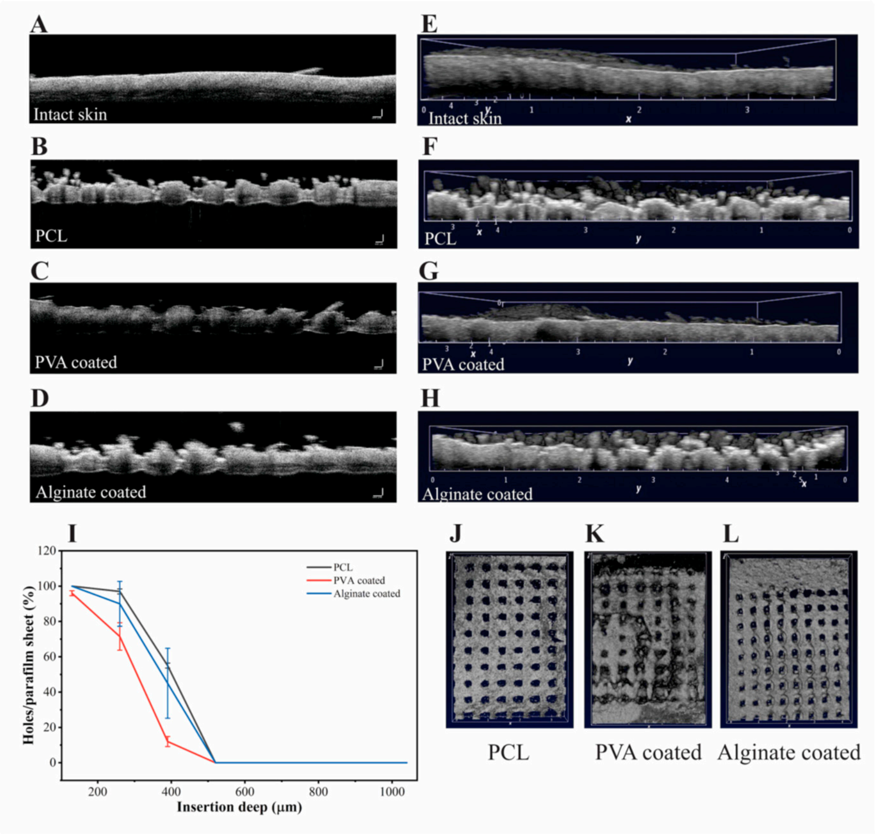

Figure S1. Parafilm® insertion efficiency of uncoated and polymer-coated PCL-MNs. The percentage of holes per Parafilm® layer was evaluated using an eight-layer Parafilm® model after application of uncoated PCL-MNs, PVA20%-PCL-MNs, and ALG4%-PCL-MNs. Representative images of perforated Parafilm® sheets are shown for each formulation. The assay was performed in triplicate and used as a complementary standardized insertion model.

Evaluating DNP-KLH integrity and stability post-release from MNs

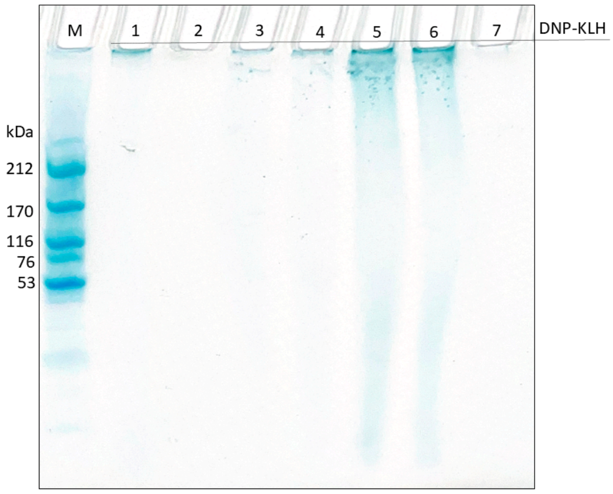

Figure S2. Structural integrity of DNP-KLH released from PVA20%-PCL MNs. SDS-PAGE analysis (4–20% gradient) of DNP-KLH recovered after 1 h release in DPBS 1X from PVA20%-PCL MNs formulations containing 10 μg or 100 μg of DNP-KLH. Lanes: M. molecular mass; 1. DNP-KLH (4 μg) was used as a reference control; 2. empty; 3-4 PVA20%-PCL MNs containing 10 μg of DNP-KLH; 5-6. PVA20%-PCL MNs containing 100 μg of DNP-KLH; 7. empty.
